# Weight of Different Intraocular Lenses: Evaluation of Toricity, Focality, Design, and Material

**DOI:** 10.1155/2021/6686700

**Published:** 2021-04-20

**Authors:** Ángel López-Vázquez, Inés Contreras, Sergio Martin-Prieto, Ángel López-Castro

**Affiliations:** ^1^Hospital Universitario Ramón y Cajal, Madrid, Spain; ^2^Hospital Universitario Ramón y Cajal, Instituto Ramón y Cajal de Investigaciones Sanitarias (IRYCIS), Clínica Rementería, Madrid, Spain; ^3^Clínica Laservision, Madrid, Spain

## Abstract

**Purpose:**

To evaluate the weight of intraocular lenses (IOLs) depending on their material, dioptric power, toricity, focality, and haptic design.

**Methods:**

Twenty-eight different IOL models from nine different medical companies (a total of 38 IOLs) and 1 capsular tension ring (CTR) were evaluated. IOLs were weighed using a precision scale, in hydrated conditions, as an approximation to their intraocular status.

**Results:**

Hydrophilic IOLs were heavier than hydrophobic lenses (*p* < 0.001). Regarding toricity, no statistical differences were found comparing toric to non-toric models (*p*=0.1). Likewise, no differences were found between multifocal IOLs and monofocal IOLs (*p*=0.19). Dioptric power did not affect IOL weight: IOLs of <15DP had similar weights to those of ≥15D and IOLs of ≥24D had similar weights to those of <24 *D* (*p*=0.86 and *p*=0.59, respectively). Plate-design IOLs were significantly heavier than 1-piece C-loop (*p* < 0.001), 3-piece C-loop (*p* < 0.001), and 4-haptic lenses (*p*=0.001).

**Conclusions:**

Of the characteristics analyzed that might influence IOL weight, lenses with hydrophilic material and plate-haptic design were found to be heavier. Toricity, focality, and dioptric power had no influence on IOL weight.

## 1. Introduction

Late in-the-bag intraocular lens (IOL) dislocation is a well-known, serious complication derived from cataract surgery. Although an exact rate has not yet been determined, a cumulative incidence of 0.1% after 10 years, 0.2% after 15 years, and 1.7% after 25 years has been reported [1], and this incidence seems to be increasing [1–3]. The proposed mechanism for dislocation is zonular insufficiency, which might be due to several factors, such as pseudoexfoliation syndrome, capsular contraction syndrome, axial myopia, previous vitreoretinal surgery, and trauma [2–7].

To the best of our knowledge, the only study on intraocular lens (IOL) weights dates back to 1977, when IOL technology was far different from that currently available [8]. IOLs are available in four types of optic material: polymethylmethacrylate (PMMA), hydrophilic acrylic, hydrophobic acrylic, and hydrophobic silicone. Hydrophobic acrylic IOLs are probably the most implanted type worldwide. Each material has advantages and disadvantages, such as different rates of posterior capsule opacification or resistance to Nd : YAG laser impacts [9]. Regarding the design of the lens, there is a wide variety available. However, 1-piece C-loop, 3-piece C-loop, and plate-design IOLs are the most frequently implanted. With the arrival of toric and multifocal IOLs, not only material or design has to be taken into account, but also the IOL's refractive properties.

Evaluating the influence of the type of lens on the risk of in-the-bag dislocation is very difficult, as it is nearly impossible to ascertain the lenses implanted in a given population, and even when there is a dislocation requiring surgery, the type of lens is difficult to determine and is often not recorded. Plate-haptic, silicone IOLs seem to produce more capsular shrinkage, one of the risk factors for dislocation; however, there have been reports of IOL dislocations for almost all IOL models [4, 7, 10–12]. Since the IOL-bag complex dislocates inferiorly in up to 90% of cases [4, 7], we believe gravity and IOL weight might influence late in-the-bag dislocation. Therefore, it would be of interest to evaluate the weight of IOLs, made from different materials and with several designs.

The main purpose of this study was to assess IOL weights depending on their optic material, dioptric power, toricity, focality, and haptic design.

## 2. Methods

**Figure 1:**
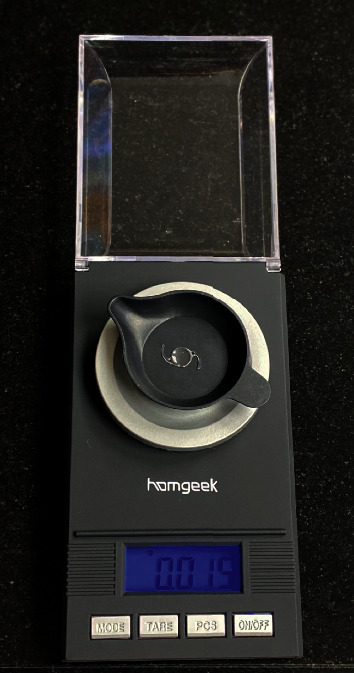
The balance used to weigh the intraocular lenses.

Twenty-eight different IOL models from nine medical companies and one PMMA capsular tension ring (CTR) were studied. They were weighed using a precision scale (Professional digital mini scale TL-series, Homgeek Incorporation, UK, precision ± 0,001 grams), in hydrated conditions (Figure 1).

**Figure 2:**
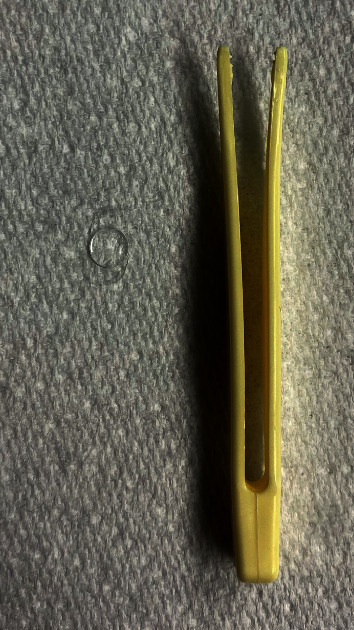
An intraocular lens, set on absorbent paper to remove surplus fluid prior to being weighed.

To simulate intraocular hydrated conditions, hydrophobic lenses and the CTR ring were immersed in 0.9% sodium chloride (B. Braun Medical AG, Melsungen, Germany) for 60 minutes. They were then weighed after drawing them out from the solution, once surface liquid surplus was removed. Hydrophilic IOLs were weighed after extraction from their package once spare conservation solution was eliminated (Figure 2). All measurements and manipulation were performed by the same blind observer (M-P, S).

Variables recorded for each IOL were dioptric spherical equivalent power (D), hydrated weight (mg), optic material, mono vs multifocality, toricity and design (1-piece C-loop, 3-piece, plate, or 4-haptic design).

### 2.1. Statistical Analysis

Descriptive statistics were calculated for all variables of interest. Mean values and standard deviations were independently calculated for all variables. The Kolmogórov–Smirnov and Shapiro–Wilk tests confirmed all numerical variables followed a normal distribution. Student's *t*-test was used with all variables except for design, on which ANOVA test was performed. Significance was set at *p* < 0.05. The SPSS software version 1.0.0.1461 (International Business Machines Corp, Armonk, NY, USA) was used for all statistical analysis.

## 3. Results

A total of 38 IOLs and one CTR were included in this study.  Table 1 records the weight and characteristics of each IOL included, and Table 2 shows average values according to the different characteristics evaluated.

The comparison between groups showed statistically significant differences regarding optic material and plate-haptic design vs other designs. The difference was higher when comparing hydrophilic versus hydrophobic lenses. Hydrophylic lenses were heavier (27.24 mg ± 4.73) compared to hydrophobic ones (17.06 mg ± 2.11), *p* < 0.01. Regarding IOL design, plate-haptic design IOLs were heavier than 1-piece C-loop and 3-piece IOLs (*p* < 0.001) and also heavier than 4-haptic design IOLs (*p* < 0.001). No statistically significant differences were found between C-loop and 4-haptic IOLs.

Although multifocal IOLs were slightly heavier than monofocal IOLs, the difference was not statistically significant (26.2 mg ± 7.09 vs 22.15 mg ± 6.16, *p*=0.19). There was also a trend for higher weights in toric lenses compared to their spherical counterparts, although again, the difference was not statistically significant (26.0 mg ± 7.09 vs 21.8 mg ± 5.96, *p*=0.1). Regarding IOL power, no significant differences were detected. IOLs with spherical equivalent ≥15D were not heavier or lighter than the group of <15D (22.77 mg ± 6.30 vs 22.29 mg ± 7.06, *p*=0.86), and the same was true for IOLs ≥24D compared to those <24D (23.86 mg ± 7.24 vs 22.42 mg ± 6.23, *p*=0.59).

## 4. Discussion

Late in-the-bag IOL dislocation is a well-known complication of cataract surgery. Of the factors which play a role in its development, most are inherent to the eye or its past medical history and thus unmodifiable: pseudoexfoliation syndrome, axial myopia, previous vitreoretinal surgery, etc [2–7]. These factors influencing the zonule's resistance have been widely studied; however, the influence of the characteristics of the IOL implanted remains unclear.

Capsular contraction syndrome derives from epithelial anterior lens cells, also known as A cells, found in the internal aspect of the anterior capsule [13]. These cells undergo a myofibroblastic differentiation when they come into contact with the IOL surface. Patients with capsular contraction syndrome are at risk of developing zonular weakness and therefore late IOL dislocation. Risk factors for developing capsular contraction syndrome are small diameter capsulorhexis, zonular weakness, chronic intraocular inflammation, pseudoexfoliation syndrome, retinitis pigmentosa, advanced age, diabetes mellitus, Behçet's syndrome, and high myopia [14]. Given that surgeons can choose the IOL to be implanted and knowing that IOL material and design have been reported as two factors influencing capsular contraction syndrome, many authors have studied their implications. It has been reported that silicone and hydrogel IOLs are associated with greater capsular contraction than acrylic models [15, 16]. As regards acrylic IOLs, hydrophilic optics have been shown to lead more frequently to capsular contraction than hydrophobic optics [15, 17]. Plate-haptic IOLs are also more frequently associated with contraction of the anterior capsule compared to other designs [17, 18]. However, all types of IOL, regardless of material or design, can dislocate over time [4, 7, 10–12]. Even though the implantation of a CTR is recommended when zonular insufficiency is suspected, as it distributes zonular tension [19, 20], in-the-bag IOL + CTR subluxation has also been frequently reported [21, 22].

As mentioned above, over 90% of late in-the-bag subluxations are inferior [7, 23], with other dislocation sites being exceptional. Therefore, we suggest that IOL weight may play a role in its pathogenesis. To our knowledge, the only report on IOL weight dates back to the 1970s, analyzing IOLs that have little in common with current IOL technology. Phacoemulsification was not performed in those days when PMMA lenses were the only available option and were implanted in many cases outside the capsular bag [8].

We found that acrylic hydrophilic IOLs were heavier than hydrophobic IOLs. Furthermore, plate-design IOLs were also significantly heavier than other designs. Toricity, focality, or IOL power does not seem to affect IOL weight. Similarly, no differences were found between the other designs included in this study (1- or 3-piece C-loop or 4-haptic).

Our study suggests that certain IOLs characteristics should be considered in patients at risk of late in-the-bag dislocation. Given that hydrophilic IOLs are significantly heavier and lead to greater shrinkage of the anterior capsule [15, 16], we propose hydrophilic IOLs should be avoided in patients with high axial myopia, pseudoexfoliation syndrome, or other risk factors if other options are available. Similarly, as plate-haptic designs were found to be significantly heavier than other designs and produce more often capsular contraction syndromes [15, 16], they should also be avoided in patients with zonular weakness. Since no differences were found when comparing mono to multifocality, toricity or non-toricity, and IOL power, it seems no precautions are necessary when considering the implantation of these lenses.

The benefits of the implantation of a CTR remain controversial. Despite having been proven to distribute zonular tension and prevent anterior capsular shrinkage [17, 18], CTR implantation can also cause an extra trauma during surgery in up to 10% of patients, especially if not adequately inserted [22]. Our study can add that if weight truly matters in intraocular implants, the extra weight of a CTR should not worry surgeons since a PMMA 11–13 mm standard CTR weighs less than 1 mg in hydrated conditions.

In conclusion, acrylic hydrophilic IOLs are significantly heavier than acrylic hydrophobic IOLs and so are plate-design IOLs compared to other designs. Knowing most late in-the-bag dislocations are inferior, heavier IOLs may lead to higher stress on zonule fibers. Therefore, in patients at risk of developing late in-the-bag IOL dislocation, acrylic hydrophilic and plate-haptic design IOLs should be avoided.

Our study has several limitations. First of all, as the anterior chamber is filled with aqueous humor, density might be a better variable to evaluate than weight. However, we do not believe so, as the IOL is supported by capsular contraction and adherence once the IOL is implanted. Second, to support our recommendations, we should know what types of IOL subluxate the most in proportion to the total implanted, something currently unknown.

## Figures and Tables

**Table 1:** Weights and characteristics of all IOLs included.

Company	Model	Se (D)	Hydrated weight (Mg)	Hydrophobic	Focality	Toric	Design
Zeiss	CT LUCIA 211P	9.50	15	Yes	Mono	No	CL
Zeiss	CT LUCIA 611P	28	19	Yes	Mono	No	CL
AJL	AIALA DRY	21	22	Yes	Mono	No	CL
Alcon	AU00T0	27	14	Yes	Mono	No	CL
Alcon	CNA0T0	18	15	Yes	Mono	No	CL
Alcon	SN60AT	7	17	Yes	Mono	No	CL
Alcon	SN60WF	18	16	Yes	Mono	No	CL
Alcon	SN60AT	18	18	Yes	Mono	No	CL
Aaren scientific	AARIS	22.50	20	Yes	Mono	No	CL
J&J	PCB00	19	17	Yes	Mono	No	CL
Topcon	LS-312	18.5	24	No	Mono	No	CL
Alcon	MN60AC	22.5	16	Yes	Mono	No	3P
Alcon	PANOPTIX	20	16	Yes	Multi	No	CL
J&J	ZA9003	11.5	20	Yes	Mono	No	3P
Zeiss	CT SPHERIS 204	21	32	No	Mono	No	P
Zeiss	CT ASPHINA 409 MP	22	31	No	Mono	No	P
Zeiss	CT ASPHINA 404	20	30	No	Mono	No	P
Topcon	LS313	20	30	No	Mono	No	P
Topcon	LS313MF30	24.5	31	No	Mono	Yes	P
Topcon	LS313 T3	10.5	29	No	Mono	Yes	P
Topcon	LS313T1	27.25	34	No	Mono	Yes	P
Zeiss	AT LISA 839 MP	12.5	26	No	Multi	No	P
Zeiss	AT LISA 939 MP	21.5	33	No	Multi	No	P
Topcon	LU313MFT	13.5	33	No	Multi	Yes	P
Medical mix	MICRO +123	17	25	No	Mono	No	4H
Medical mix	MICRO +A123	25.5	26	No	Mono	No	4H
Medical mix	MICROPURE 123	27.5	18	No	Mono	No	4H
Medical mix	MICROPURE 123	12.5	16	No	Mono	No	4H
Medical mix	FINEVISION PO	15	23	No	Multi	No	4H
Medical mix	ANKORIS TORIC	20.25	24	No	Mono	Yes	4H
Medical mix	ANKORIS TORIC	24.75	25	No	Mono	Yes	4H
Medicontur	BI-FLEX	19.5	28	No	Mono	No	CL
Medicontur	BI-FLEX	18.5	26	No	Mono	No	CL
Medicontur	BI-FLEX	19.5	28	No	Mono	No	CL
B&L	MX60T	20.62	16	1Yes	Mono	Yes	CL
B&L	MX60T	20.62	16	1Yes	Mono	Yes	CL
B&L	MX60T	21	16	1Yes	Mono	Yes	CL
B&L	MX60T	22	17	1Yes	Mono	No	CL
Freedom ring	CTR 13-11	0	<1				

Mono, monofocal; Multi, multifocal; CL, C-loop; 4H, four haptics; P, plate; 3P, 3-piece.

**Table 2:** Average values according to the different characteristics evaluated.

Group	n	Average weight (mg)	Standard deviation
Hydrophobic	17	17.06	2.11
Hydrophilic	21	27.24	4.73
Monofocal	33	22.15	6.16
Multifocal	5	26.20	7.19
Toric	8	26.00	7.09
Non-toric	30	21.80	5.96
<15 D	7	22.29	7.06
≥15 D	31	22.77	6.30
≥24 D	7	23.86	7.24
<24 D	31	22.42	6.23
C-loop	19	18.95	4.48
4-haptic	7	22.43	3.87
Plate	10	30.90	2.33
3-piece	2	18	2.83

## Data Availability

The IOL weight data used to support the findings of this study are included within the article.
